# Buildings Contemplated and in Progress

**Published:** 1911-06-24

**Authors:** 


### Hospital Architecture and Construction.

[Communications on this subject should be marked "Architecture" in the left-hand top corner of the envelope.]

### BUILDINGS CONTEMPLATED AND IN PROGRESS.

Bradford.—Fifty-four designs have been submitted in open competition for the new infirmary. The President of the Royal Institute of British Architects has nominated Mr. Keith Young, of London, as assessor. The building fund has already reached close on £80,000.

Bingley, Yorks.—The committee of the Bingley Cottage Hospital has decided to add a new ward at an estimated cost of £700. This will double the present accommodation, and if the necessary capital is forthcoming it is proposed to name the extension "The King's Ward."

Bucknall.—It is proposed to erect an isolation hospital, at a cost of £2,350, by the Stoke-on-Trent and Stoke Rural Joint Hospital Board.

Newcastle-on-Tyne.—Extensions are to be made to the hospital. Particulars may be obtained from the clerk to the Board of Guardians.

Orrel.—A smallpox hospital is to be built at Orrel, and information regarding it may be had from the surveyor to the Blackrod Urban District Council.

Pontypridd (Llwynypia).—The new workhouse infirmary, designed by Messrs. Evans, has been formally opened. It comprises nine wards, each with twelve beds; three of the wards are for consumptive cases. Two large boilers supply low pressure for heating, hot water, and cooking.

Stockport.—Mr. James Jepson, of Stockport, architect for the extension of Dailstone Lane Hospital, will send quantities to builders on deposit of £3 3s. Tenders are to be returned before June 27.

Swaffham.—Plans and specifications have been prepared for the alteration to the hospital, including a new operating-room, and the papers may be had from Mr. J. A. Gould, of Swaffham.

Swansea.—Under the superintendence of Mr. Glendinning Moxham, F.R.I.B.A., extensions are being carried out at the Swansea Hospital, including casualty department, dispensary, nurses' home, wards containing eighty beds, modernising administrative and staff quarters, and the erection of an ophthalmic theatre.

Warwickshire.—Mr. West, County Hall, Warwick, is architect for the proposed sanatorium at Binton Hill.

### PRIZEWINNERS IN THE BRADFORD INFIRMARY COMPETITION.

As we go early to press we learn the results of the Bradford Infirmary competition in connection with the architects' competition for the best design of the proposed new Royal Infirmary at Bradford. Competitive plans were sent in by forty-four architects. These were adjudicated upon by Mr. Keith D. Young, F.R.I.B.A., the assessor appointed by the Board of Management, and he has now announced his award as follows :—

*First Prize*, £150.—Design No. 35 (Mr. Wm. A. Pite, F.R.I.B.A., 116 Jermyn Street, St. James's, London).

*Second Prize*, £100.—Design No. 8 (Messrs. F. E. Halford and A. T. Cutler, 8 Southampton Street, London, W.C.).

*Third Prize*, £50.—Design No. 24 (Messrs. Arthur Marshall, P. D. Prior, and W. A. Smith, Russell Chambers, Nottingham).

